# Six-Year Follow-Up of Impact of Co-proxamol Withdrawal in England and Wales on Prescribing and Deaths: Time-Series Study

**DOI:** 10.1371/journal.pmed.1001213

**Published:** 2012-05-08

**Authors:** Keith Hawton, Helen Bergen, Sue Simkin, Claudia Wells, Navneet Kapur, David Gunnell

**Affiliations:** 1 Centre for Suicide Research, University of Oxford Department of Psychiatry, Warneford Hospital, Headington, Oxford, United Kingdom; 2 Office for National Statistics, Government Buildings, Newport, United Kingdom; 3 Centre for Suicide Prevention, University of Manchester, Manchester, United Kingdom; 4 School of Social and Community Medicine, University of Bristol, Bristol, United Kingdom; Institute of Psychiatry, King's College London, United Kingdom

## Abstract

**Background:**

The analgesic co-proxamol (paracetamol/dextropropoxyphene combination) has been widely involved in fatal poisoning. Concerns about its safety/effectiveness profile and widespread use for suicidal poisoning prompted its withdrawal in the UK in 2005, with partial withdrawal between 2005 and 2007, and full withdrawal in 2008. Our objective in this study was to assess the association between co-proxamol withdrawal and prescribing and deaths in England and Wales in 2005–2010 compared with 1998–2004, including estimation of possible substitution effects by other analgesics.

**Methods and Findings:**

We obtained prescribing data from the NHS Health and Social Care Information Centre (England) and Prescribing Services Partneriaeth Cydwasanaethau GIG Cymru (Wales), and mortality data from the Office for National Statistics. We carried out an interrupted time-series analysis of prescribing and deaths (suicide, open verdicts, accidental poisonings) involving single analgesics. The reduction in prescribing of co-proxamol following its withdrawal in 2005 was accompanied by increases in prescribing of several other analgesics (co-codamol, paracetamol, codeine, co-dydramol, tramadol, oxycodone, and morphine) during 2005–2010 compared with 1998–2004. These changes were associated with major reductions in deaths due to poisoning with co-proxamol receiving verdicts of suicide and undetermined cause of −21 deaths (95% CI −34 to −8) per quarter, equating to approximately 500 fewer suicide deaths (−61%) over the 6 years 2005–2010, and −25 deaths (95% CI −38 to −12) per quarter, equating to 600 fewer deaths (−62%) when accidental poisoning deaths were included. There was little observed change in deaths involving other analgesics, apart from an increase in oxycodone poisonings, but numbers were small. Limitations were that the study was based on deaths involving single drugs alone and changes in deaths involving prescribed morphine could not be assessed.

**Conclusions:**

During the 6 years following the withdrawal of co-proxamol in the UK, there was a major reduction in poisoning deaths involving this drug, without apparent significant increase in deaths involving other analgesics.

*Please see later in the article for the Editors' Summary*

## Abstract 2

A time-series study conducted by Keith Hawton and colleagues reports on the links between withdrawal of the analgesic co-proxamol and subsequent prescribing and deaths associated with analgesic poisoning.

## Editors' Summary

**Background:**

Every year, about a million people worldwide die by suicide. Most people who take their own life are mentally ill. For some, stressful events have made life seem worthless or too painful to bear. Suicide rates can be reduced by improving the treatment of mental illness and of stress and by limiting access to common suicide methods. These methods differ from place to place. For example, in England and Wales, where 4,528 suicides were recorded in 2010, drug-related poisoning is responsible for about a fifth of all suicides. Notably, between 1997 and 1999, the prescription analgesic (pain killer) co-proxamol, which contains paracetamol and the opioid dextropropoxyphene, was implicated in a fifth of drug-poisoning suicides in England and Wales. In response to concerns about co-proxamol's widespread use for suicidal poisoning and its safety/effectiveness profile, the UK Committee on Safety of Medicines announced on January 31, 2005 that the drug would be withdrawn completely from use in the UK by December 31, 2007, and that between 2005 and 2007, doctors should not prescribe co-proxamol to any new patients and should try to move patients already taking the drug onto other medications.

**Why Was This Study Done?:**

Public health experts need to quantify the impact of co-proxamol withdrawal on analgesic prescribing in England and Wales and on suicide rates. In particular, they need to know whether its withdrawal has increased the use of other analgesics in suicide (substitution). Reassuringly, between 2005 and 2007, there was a reduction in both co-proxamol prescribing and in co-proxamol–related suicides in England and Wales but no evidence of increased poisoning deaths involving other prescription analgesics. But what about the longer-term effects of co-proxamol withdrawal? In this interrupted time-series study, the researchers assess the impact of co-proxamol withdrawal in England and Wales by comparing data on analgesic prescribing and suicide rates collected between 1998 and 2004 and between 2005 and 2010. An interrupted time-series study uses serial measurements of events in a population before and after an intervention to look for changes in response to the intervention.

**What Did the Researchers Do and Find?:**

The researchers obtained prescribing data from 1998 to 2010 for co-proxamol and several other analgesics from government sources in England and Wales, and data on suicides, open verdicts (poisoning of undetermined intent), and accidental poisonings involving single analgesics from the UK Office for National Statistics. They then estimated changes in levels and trends in prescribing and deaths following the 2005 announcement of co-proxamol withdrawal using interrupted time-series analysis. There was a marked reduction in co-proxamol prescribing that was accompanied by increased prescribing of several other analgesics after co-proxamol withdrawal. These changes were associated with a major reduction in suicide deaths due to poisoning that equated to 500 fewer deaths occurring between 2005 and 2010 than would have occurred had co-proxamol not been withdrawn. On average, there were 20 co-proxamol-related deaths (suicides and accidental poisonings) per year during 2008–2010 compared to more than 250 per year in the 1990s. Finally, there was little evidence of a change in the number of deaths involving other analgesics after co-proxamol withdrawal except for a small increase in the number of deaths involving the opioid oxycodone.

**What Do These Findings Mean?:**

These findings show that, during the six years that followed the beginning of the phased withdrawal of co-proxamol in the UK, there has been a major reduction in poisoning deaths involving this drug in England and Wales. The findings provide little evidence for an increase in deaths involving other analgesics. However, because the Office of National Statistics does not distinguish between deaths due to oral and intravenous morphine or between deaths due to morphine and heroin, the researchers did not assess whether there have been any changes in deaths involving morphine since 2005. Moreover, this study did not assess suicides related to the use of multiple drugs or investigate whether suicides involving methods other than drug-related poisoning have increased since co-proxamol withdrawal. Despite these limitations, these findings suggest that the withdrawal of co-proxamol in the UK and possibly elsewhere should have major beneficial effects on suicide rates, at least in the relatively short term.

**Additional Information:**

Please access these Web sites via the online version of this summary at http://dx.doi.org/10.1371/journal.pmed.1001213.

- The World Health Organization provides information on the global burden of suicide and on suicide prevention (in several languages)
- The US National Institute of Mental Health provides information on suicide and suicide prevention web page
- The UK National Health Service Choices website has detailed information about suicide and its prevention
- The University of Oxford Centre for Suicide Research provides information about co-proxamol and suicide and links to other research on suicide
- The 2005 announcement of the withdrawal of co-proxamol in the UK is available through the UK Medicines and Healthcare products Regulatory Agency, which also provides a question and answer document about the risks and benefits of co-proxamol
- MedlinePlus provides links to further resources about suicide (in English and Spanish)
- The charity Healthtalkonline has personal stories about dealing with suicide
- Help is at Hand is a resource for people bereaved by suicide

## Introduction

In January 2005, following a review of the effectiveness and safety profile of the analgesic co-proxamol by the UK Medicines and Healthcare products Regulatory Agency (MHRA), the UK Committee on Safety of Medicines (CSM) announced that the drug should be withdrawn from use in the UK, the final date of withdrawal being 31st December 2007. During the intervening period (2005–2007), doctors were advised not to prescribe co-proxamol to any new patients and to make efforts to move patients to suitable alternative medication (although patients for whom this was difficult could continue to receive the drug through normal prescribing) [1],[2]. The alternatives suggested for acute pain were, first, paracetamol, secondly, the non-steroidal anti-inflammatory drug (NSAID) ibuprofen, thirdly, a combination of the two, fourthly, paracetamol and an alternative NSAID (with codeine or dihydrocodeine being alternatives to NSAIDs where these were contraindicated). Similar steps were recommended for chronic pain, but with possible addition of codeine or dihydrocodeine to paracetamol and an NSAID, plus use of a tricyclic antidepressant or anticonvulsant where pain control was difficult. Where the chronic pain was progressive, for example cancer, more potent opioids (e.g., morphine, oxycodone) could be considered [1].

The background to these steps was longstanding concern about the extent of fatal self-poisoning with co-proxamol [3],[4]. The drug is a combination of paracetamol (acetaminophen) and the opiate dextropropoxyphene. Death following overdose usually occurs because of the toxic effects of high levels of dextropropoxyphene on respiration and cardiac conduction [5],[6]. There is a relatively narrow margin between therapeutic and potentially lethal concentrations [7]. While concerns had been expressed over many years about the number of deaths involving co-proxamol [3],[8], the MHRA review was prompted by research that showed that between 1997 and 1999, co-proxamol was the single drug used most frequently for suicide in England and Wales (with 766 deaths over the 3-y period) and was implicated in a fifth of all drug-poisoning suicides, or approximately 5% of all suicides [4]. The MHRA also had concerns about limited efficacy of co-proxamol, safety in therapeutic use, and abuse potential [3],[9].

In June 2009, following the UK lead, the European Medicines Agency recommended that dextropropoxyphene-containing medication be withdrawn throughout the European Union [10], which became European policy in June 2010, with full withdrawal by September 2011. In 2009, a US Foods and Drugs Administration (FDA) panel recommended that the FDA should withdraw dextropropoxyphene from the US market [11], and in 2010 the FDA instructed manufacturers to cease production [12]. Also, in 2010 all dextropropoxyphene products were withdrawn in Canada [13].

During the 3-y withdrawal phase in the UK (2005–2007), a major reduction in prescribing of co-proxamol was recorded in both England and Wales [14]–[16] and Scotland [17]. Beneficial effects on suicides involving co-proxamol were shown in Scotland for 2005–2006 [17] and in England and Wales for 2005–2007 [16]. Importantly, in England and Wales no evidence of increased poisoning deaths involving other analgesics was found, in spite of significantly greater prescribing of some preparations, specifically co-codamol, paracetamol, co-dydramol, and codeine. The changes in England and Wales were associated with fewer suicidal and accidental poisoning deaths involving co-proxamol over the 3-y period [16]. In Ireland, where co-proxamol (Distalgesic) was totally withdrawn in January 2006, there was a very marked reduction in intentional non-fatal co-proxamol overdose presentations to general hospitals between 2006 and 2008, which greatly exceeded the increase seen in non-fatal overdoses of other analgesics [18]. A similar finding was shown for non-fatal self-poisoning presentations to general hospitals in England between 2005 and 2007, although there was an upturn in episodes involving other analgesics in 2007 [19]. Thus the initial withdrawal phase in the UK, and the total withdrawal in Ireland, appeared not only to result in a major reduction in poisoning deaths and non-fatal overdoses involving co-proxamol, but also only limited substitution by fatal or non-fatal poisonings with other analgesics.

It is important to examine longer-term effects of the initiative in order to determine the impact of the full withdrawal of co-proxamol and, especially, whether there has been a substitution effect in terms of a gradual move to use of other dangerous methods of self-poisoning [20]. The aim of the present study was to evaluate the longer-term impact of the withdrawal of co-proxamol in England and Wales, including the initial withdrawal phase (2005–2007) and the first 3 y of the full withdrawal period (2008–2010). Our evaluation has included assessment of the impact on prescribing of analgesics and on poisoning deaths involving co-proxamol and other analgesics. While our main focus was on suicidal poisonings, we have also included accidental poisoning deaths.

## Method

### Prescriptions

Quarterly data for 1998–2010 on prescriptions of co-proxamol, co-codamol, codeine, co-dydramol, dihydrocodeine, NSAIDs, paracetamol, and tramadol in England and Wales were obtained from the NHS Health and Social Care Information Centre (England) and Prescribing Services Partneriaeth Cydwasanaethau GIG Cymru (Wales). Prescription data for Wales were not available for the first quarter of 1998, so figures for this quarter were estimated using least squares methods to extrapolate from trend data for subsequent quarters.

We also obtained prescription data for oral morphine and for oxycodone for England only between 1998 and 2010 (data for Wales were unavailable for some of this period).

### Deaths

Quarterly data on drug-poisoning deaths (suicides, open verdicts, and accidental poisonings) involving co-proxamol, co-codamol, codeine, co-dydramol, dihydrocodeine, NSAIDs, paracetamol, and tramadol based on death registrations during 1998–2010 in England and Wales were provided by the Office for National Statistics (ONS) (we also received annual death data for oxycodone). Data were extracted from the ONS database of deaths related to drug poisoning, which is extracted from the national mortality database for England and Wales. Deaths are included if the underlying cause of death is regarded as drug related, according to the National Statistics definition [21]. Almost all deaths on the drug-poisoning database had a coroner's inquest. For each death the database includes the underlying cause of death, all other causes mentioned on the death certificate, and every mention of a substance recorded by the coroner in the cause of death section or elsewhere in the coroner's certificate produced after the inquest. For compound analgesics all the components of the compound are mentioned, with the exception of dextropropoxyphene, when co-proxamol is coded whether or not paracetamol is mentioned, because in the UK dextropropoxyphene is rarely, if ever, available other than as part of a paracetamol compound.

Analyses were restricted to deaths involving single drugs, or single drugs and alcohol. Similar data were supplied for overall drug-poisoning deaths receiving underlying causes of death of suicide, undetermined, or accidental poisoning. These data are based on the coroners' short form verdicts and, in the case of narrative verdicts (where an account of the death is provided rather than a short form verdict), on International Classification of Disease (ICD) coding rules. Coroners reach their verdicts on the basis of information from clinicians, relatives, and police, and, in the case of poisonings, on findings of toxicological reports. In a proportion of injury and poisoning deaths where a narrative verdict has been returned, ONS has no indication from the information provided by the coroner of whether the toxic substance was self-administered or if there was deliberate intent to self-harm. Such deaths are coded as accidental in line with ICD coding rules [22].

In order to evaluate the impact of the withdrawal of co-proxamol on suicide, we have used data on deaths receiving a suicide verdict and those coded as injury or poisoning of undetermined intent (open verdicts) by the ONS. In England and Wales, it has been customary to assume that most of the latter are cases where the harm was self-inflicted but there was insufficient evidence to prove intent to die [23],[24].

### Statistical Analyses

Analyses of trends in prescribing and deaths were conducted using Stata version 10.0 [25]. We used interrupted time-series analysis to estimate changes in levels and trends in prescribing and deaths following the CSM announcement of the withdrawal of co-proxamol. This method controls for baseline level and trend when estimating expected changes in the number of prescriptions (or deaths) due to the intervention [26].

Specifically, segmented regression analysis [27] was used to estimate the mean quarterly number of prescriptions and deaths that might have occurred in the post-intervention period without the CSM announcement, and the number of prescriptions and deaths that actually occurred following the CSM announcement. The latter were obtained from best fitted data lines from the regressions and are better estimates than taking the average of the actual values. The beginning of 2005 was chosen as the point of intervention. Thus our data comprised 28 quarters in the pre-intervention segment and 24 quarters in the post-intervention segment. Slope and level regression coefficients were used to estimate the mean quarterly absolute differences in prescriptions and deaths (at the midpoint of the post-intervention period, midway between quarter 4 of 2007 and quarter 1 of 2008).

Preliminary analyses indicated some autocorrelation in the data, therefore the Cochrane-Orcutt autoregression procedure was used (rather than ordinary linear regression) to correct for first order serially correlated errors. The Durbin Watson statistic of all final models was close to the preferred value of 2, indicating that no serious autocorrelation remained.

## Results

### Prescriptions for Co-proxamol and Other Analgesics

**Figure 1:**
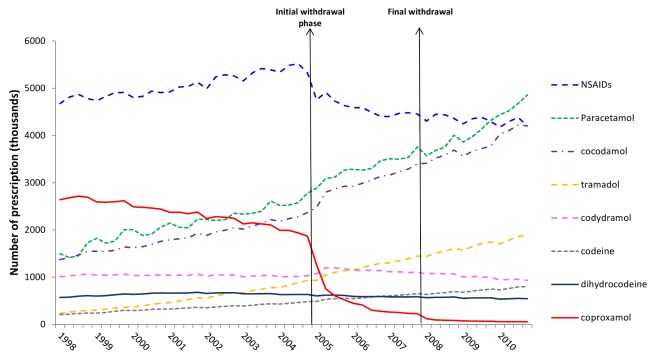
Trends in prescriptions dispensed for co-proxamol and seven other analgesics in England and Wales, 1998–2010.

As expected, prescribing of co-proxamol showed a marked reduction after the beginning of the initial withdrawal. Thus there was a step change reduction in prescribing of co-proxamol in England and Wales during 2005–2010 compared to the trend in prescribing during 1998–2004 (Figure 1). At the beginning of 2008 there was a further downward step in prescribing such that there were very few prescriptions during 2008–2010. Overall between 2005 and 2010 there was a 53% reduction in prescribing of co-proxamol compared to previous trends (Table 1). There were significant increases in prescribing of co-codamol (+23%), paracetamol (+16%), codeine (+10%), co-dydramol (+6%), and tramadol (+19%) during 2005–2010 (Figure 1; Tables 1 and S1). There was a sharp decrease in prescribing of NSAIDs, which began shortly before the withdrawal of co-proxamol (which was due to safety concerns about Cox2 inhibitors [28]). There was also a decrease in prescriptions of dihydrocodeine (−10%) in 2005–2010. Overall, when all seven analgesics (excluding co-proxamol) were combined this showed no significant change in either level or trend associated with the withdrawal of co-proxamol. With NSAIDs excluded, however, there was a significant 15% increase in prescribing of the other six analgesics combined.

**Table 1:** Changes in prescriptions involving co-proxamol and seven other analgesics in England and Wales, 1998–2010, associated with the Committee on Safety of Medicines (CSM) announcement in January 2005.

Prescriptions (Thousands)	Estimation of the Absolute Effect during 2005–2010 of the CSM Announcement^a^			
	Mean Quarterly Estimated Number without CSM Announcement^b^	Mean Quarterly Estimated Number with CSM Announcement^b^	Mean Quarterly Change during 2005 to 2010^c^	(95% CIs)^d^
Co-proxamol	1,018	482	−536	−941 to −130
Co-codamol	2,762	3,402	640	554 to 726
Codeine	591	648	57	44 to 70
Co-dydramol	1,014	1,074	60	36 to 85
Dihydrocodeine	643	580	−63	−105 to −22
NSAIDs	5,794	4,453	−1,341	−1,507 to −1,176
Paracetamol	3,222	3,745	523	288 to 759
Tramadol	1,207	1,440	233	179 to 288
Seven analgesics other than co-proxamol	10,104	10,207	103	−132 to 337
Six analgesics other than co-proxamol and NSAIDs	9,436	10,889	1,453	1,200 to 1,706

a Using interrupted time-series segmented regression analysis where the intervention point is taken as the end of 2004 (the CSM announcement on the withdrawal of co-proxamol, January 2005).

b Estimated for the midpoint quarter of 2005–2010. See Text S1 for method, equation (2) or (3).

c Absolute difference of estimated number with CSM announcement and estimated number without CSM announcement, taken at the mid-point of the post-intervention period, see Text S1 equation (4).

d 95% CIs taken from Stata results or calculated according to Zhang et al. [38].

Prescription data for morphine were only available for England for the study period. Our analysis showed a significant mean quarterly increase in the number of prescriptions of morphine associated with the CSM announcement of 71,000 prescriptions (95% CI 62,000–79,000), equating to a 35% increase in the period 2005 to 2010. Prescription data for oxycodone were also only available for England for the study period. The number of prescriptions increased steadily from very low levels (mean of 69,000 per year in 1998–2004) to a mean of 527,000 per year in 2005–2010. An interrupted time-series analysis, as was conducted on other prescription data, did not generate meaningful estimates because of small numbers.

### Deaths Involving Co-proxamol and Other Analgesics

The numbers of deaths with suicide (including open verdicts) or accidental poisoning verdicts between 2000 and 2010 involving co-proxamol alone and those involving the seven other analgesics alone (both with or without alcohol), are shown in Table 2, together with all drug-poisoning deaths and all suicides. Between 2005 and 2010 there was a marked reduction in the numbers and proportions of all poisoning deaths recorded as suicide that involved co-proxamol. While the numbers of deaths involving other analgesics receiving a suicide or open verdict also declined, they showed a small increase in percentage of the total number of poisoning deaths. There was a similar marked decrease in deaths involving co-proxamol when accidental deaths were included, but without an increase in percentage of poisoning deaths involving other analgesics (although numbers of accidental poisonings with other analgesics increased slightly in 2008–2010). Exclusion of NSAIDs made little difference to the results of these analyses.

**Table 2:** Suicide and open verdict deaths by all causes, and suicide, open verdict, and accidental deaths due to poisoning by all drugs, co-proxamol alone, and seven other analgesics alone (or with alcohol) in England and Wales, 1998–2010.

Year	All Causes	All Drugs		Co-proxamol Alone		Other Analgesics^a^ Alone	
	Suicide, Open	Suicide, Open	Suicide, Open, Accidental	Suicide, Open	Suicide, Open, Accidental	Suicide, Open	Suicide, Open, Accidental
				*n* (%)^b^	*n* (%)^b^	*n* (%)^b^	*n* (%)^b^
1998	5,347	1,432	2,246	259 (18.1)	309 (13.8)	202 (14.1)	277 (12.3)
1999	5,241	1,414	2,294	260 (18.4)	316 (13.8)	207 (14.6)	279 (12.2)
2000	5,081	1,309	2,143	261 (19.9)	303 (14.1)	175 (13.4)	230 (10.7)
2001	4,904	1,279	2,180	232 (18.1)	276 (12.7)	195 (15.2)	257 (11.8)
2002	4,762	1,225	1,983	204 (16.7)	247 (12.5)	182 (14.9)	237 (12.0)
2003	4,811	1,194	1,843	188 (15.7)	218 (11.8)	166 (13.9)	237 (12.9)
2004	4,883	1,246	2,006	189 (15.2)	230 (11.5)	168 (13.5)	233 (11.6)
2005	4,718	1,154	1,926	131 (11.4)	157 (8.2)	196 (17.0)	254 (13.2)
2006	4,513	979	1,821	67 (6.8)	81 (4.4)	200 (20.4)	287 (15.8)
2007	4,322	888	1,852	52 (5.9)	61 (3.3)	151 (17.0)	209 (11.3)
2008	4,603	884	2,071	29 (3.3)	34 (1.6)	157 (17.8)	257 (12.4)
2009	4,682	898	2,185	21 (2.3)	26 (1.2)	155 (17.3)	267 (12.2)
2010	4,528	873	2,137	8 (0.9)	10 (0.5)	150 (17.2)	257 (12.0)

a Other analgesics: co-codamol, codeine, co-dydramol, dihydrocodeine, NSAIDS, paracetamol, and tramadol.

b Percentage of all drug-poisoning deaths.

**Figure 2:**
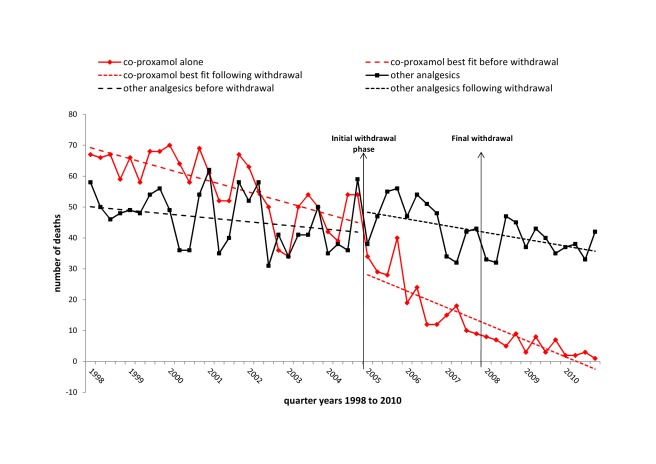
Deaths in England and Wales from poisoning with co-proxamol and other analgesics, 1998–2010. Suicide and open verdicts, substances taken alone, with or without alcohol.

The impact of the initial withdrawal phase (2005–2007) and of the full withdrawal (2008–2010) on deaths involving co-proxamol and deaths involving other analgesics are shown graphically in Figure 2. There was some reduction in deaths involving co-proxamol in the 2 or 3 y preceding the beginning of the initial withdrawal phase (2005–2007), then a steady marked reduction during the initial withdrawal phase (2005–2007), with a further smaller reduction during the first 3 y of the full withdrawal (2008–2010). The number of deaths involving other analgesics did not appear to change markedly during these periods.

The overall apparent beneficial changes following the withdrawal of co-proxamol were confirmed when the data were subjected to interrupted time series regression analyses (Table 3). During the 2005–2010 period there was a highly significant mean quarterly reduction in deaths involving co-proxamol alone that received a suicide (including open) verdict (−21 [95% CI −34 to −8]), and similarly when accidental deaths were included (−25 [95% CI −38 to −12]). There was no significant change in deaths involving the seven other analgesics combined. When we examined these drugs separately (see Table S1) none showed a significant step or trend change associated with the withdrawal of co-proxamol. There were increases in deaths involving co-codamol and codeine during the overall study period, but these began well before the withdrawal of co-proxamol. All drug-poisoning deaths (excluding those involving co-proxamol and other analgesics) receiving a suicide or open verdict were reduced following the withdrawal of co-proxamol, although when accidental deaths were included they showed a significant increase of 84 (95% CI 28–141) per quarter (Table 3).

**Table 3:** Changes in poisoning deaths involving co-proxamol, other analgesics, and all drugs, in England and Wales, 1998–2010, associated with the Committee on Safety of Medicines (CSM) announcement in January 2005.

Cause of Death	Estimation of the Absolute Effect during 2005 to 2010 of the CSM Announcement^a^			
	Mean Quarterly Estimated Number without CSM Announcement^b^	Mean Quarterly Estimated Number with CSM Announcement^b^	Mean Quarterly Change during 2005 to 2010^c^	(95% CIs)^d^
**Suicide, Open**				
Co-proxamol	34	13	−21	−34 to −8
Other analgesics^e^	38	42	4	−8 to 16
Other analgesics (except NSAIDs)^e^	38	40	2	−10 to 13
All drugs (except co-proxamol and other analgesics)	201	181	−19	−49 to 10
All drugs	275	236	−39	−87 to 9
All causes	1,129	1,140	11	−81 to 104
**Suicide, Open, Accidental**				
Co-proxamol	40	15	−25	−38 to −12
Other analgesics^e^	55	63	8	−7 to 24
Other analgesics (except NSAIDs)^e^	54	61	7	−7 to 21
All drugs (except co-proxamol and other analgesics)	336	420	84	28 to 141
All drugs	430	499	69	−7 to 144

a Using interrupted time-series segmented regression analysis where the intervention point is taken as the end of 2004 (the CSM announcement on the withdrawal of co-proxamol, January 2005).

b Estimated for the midpoint quarter of 2005–2010. See Text S1 for method, equation (2) or (3).

c Absolute difference of estimated number with CSM announcement and estimated number without CSM announcement, taken at the mid-point of the post-intervention period, see Text S1 equation (4).

d 95% CIs taken from Stata results or calculated according to Zhang et al. [38].

e Other analgesics include: co-codamol, codeine, co-dydramol, dihydrocodeine, NSAIDS, paracetamol, and tramadol.

The estimated reduction in deaths involving co-proxamol alone between 1998–2004 and 2005–2010 was 61% for suicide and open verdict deaths and 62% when accidental deaths were included. This reduction equated to approximately 500 fewer deaths from suicide between 2005 and 2010 than would be expected without the withdrawal, and 600 fewer deaths when accidental poisonings were included. As can be seen in Table 2, during the full withdrawal phase (2008–2010) there was an average of just 19 deaths per year (23 per year including accidents). This figure is in contrast to 228 deaths per year during 1998–2004 (271 per year including accidents).

We have not presented data on deaths involving morphine because ONS cannot distinguish between deaths due to oral and intravenous administration (and between those due to morphine or heroin). Deaths involving oxycodone alone and receiving suicide, open, or accidental verdicts increased during the study period from a mean of 2.3 per year between 2001 and 2004 to a mean of 8.2 per year between 2005 and 2010 (including 15 in 2010).

## Discussion

We previously demonstrated an apparent beneficial effect of withdrawal of co-proxamol in the UK in terms of deaths (suicides, open verdicts, and accidents) in England and Wales during the initial 3-y withdrawal phase (2005–2007) [16]. We have now assessed the impact of the withdrawal over a 6-y period, including the initial phase and the first 3 y of full withdrawal, against trends for the 7 y prior to the announcement of the withdrawal. This investigation has shown further significant changes following the withdrawal, not only in terms of the expected reduced prescribing of co-proxamol, but also increased prescribing of some other analgesics suggested by the CSM as substitutes for co-proxamol (paracetamol, co-codamol, codeine, co-dydramol, and, for progressive chronic pain, oxycodone and morphine), and also tramadol. During 2005–2010 there was a 61% reduction in deaths due to co-proxamol poisoning, equating to approximately 500 fewer deaths receiving suicide or open verdicts, and 600 when accidental poisonings are included. Some of the accidental poisonings are also likely to have been probable suicides, especially since there has been a recent increase in narrative verdicts by coroners, and where those responsible for coding cause of death at the ONS have difficulty in deciding the cause from the narrative, they are obliged by international convention to record the death as accidental [22]. Most importantly, the major reduction in deaths involving co-proxamol was not associated with a compensatory overall increase in deaths due to poisoning with other analgesics (although the number of accidental poisonings with other analgesics increased in 2008–2010).

During the full withdrawal phase prescribing of co-proxamol did not cease completely, presumably because of off-licence prescribing. Also, deaths from poisoning with co-proxamol did not reach zero (although there were just eight suicide deaths in 2010, and ten including accidental poisonings), partly again possibly because of off-licence prescribing, but also to residual supplies remaining in homes, and, possibly, because of supplies being obtained through internet sources. The acute reduction in prescribing of NSAIDs that began just before the withdrawal of co-proxamol, due to concerns about COX 2 inhibitors [28], would have been unlikely to have affected the findings as NSAIDs alone are rarely implicated in poisoning deaths, especially by suicide [29].

There was an overall reduction in poisoning deaths receiving suicide or open verdicts in England and Wales in 2005–2010, although this did not reach statistical significance. However, suicides overall were not reduced, possibly because of the effects of the recession that began in 2008 [30],[31]. The increase in overall poisoning deaths during 2005–2010 appears to be related to increased numbers of deaths involving methadone, and also benzodiazepines usually combined with other drugs [21]. Increased use of narrative verdicts, as noted above, may also have contributed.

The apparent overall beneficial effects of the withdrawal of co-proxamol in the UK are in keeping with the principle of reducing access to means for suicidal acts as a key strategy in suicide prevention [20]. The crucial question is whether withdrawal of a common lethal means of suicide is associated with substitution by other methods that nullify any beneficial effects. Other studies examining effects of restriction of access to means for suicidal acts have suggested that major substitution by other methods does not necessarily occur [32],[33]. Our results similarly indicate that no significant substitution followed the UK initiative and hence this appears to represent a success story in terms of suicide prevention. The impact of the withdrawal of co-proxamol should, however, be monitored further to see whether there is any compensatory increase in deaths involving other analgesics in the longer term.

There have been other changes in availability of analgesics that could possibly be relevant to this evaluation. One is the limitations on pack sizes of paracetamol, legislation on which came into force in the UK in 1998 [34]. While this measure appears to have had an impact on the size of paracetamol overdoses, deaths, and liver unit activity [29],[35], it is unlikely to have affected the impact of the withdrawal of co-proxamol as this occurred much later. Another change has been the increased prescribing of opiates, especially morphine and oxycodone. Unfortunately ONS is unable to distinguish deaths due to intravenous administration of morphine from oral administration (nor deaths due to morphine and heroin because both drugs share the same breakdown pathway). It is therefore not possible to know whether recent increased prescribing of morphine could have influenced the findings. There has been a recent increase in poisoning deaths involving oxycodone, but the numbers involved are relatively small so this is unlikely to affect the general conclusions of this study. Oxycodone is increasingly a drug of abuse [36], and hence some deaths may be unintentional. Importantly, neither morphine or oxycodone were recommended as first- or second-line analgesics to replace co-proxamol, only as drugs that might be used in chronic progressive pain, as in cancer patients [1].

### Strengths and Limitations

A strength of the study is that it is based on national data and includes reasonably long pre-intervention (7-y) and post-intervention (6-y) periods. Also, the method of statistical analysis (interrupted time-series regression) controls for baseline trends when estimating effects of the intervention on prescribing and deaths, which is preferable to methods such as comparison of changes in proportions before and after the intervention. However, estimates of overall effects do involve extrapolation and hence are subject to some degree of uncertainty. Also, percentage changes during the post-intervention period compared with the pre-intervention period are based on mean quarterly change estimates, which have associated uncertainty, and therefore should be interpreted with caution.

In order to ensure that we were examining deaths due to specific drugs we based our mortality calculations solely on deaths involving single analgesics alone (with or without alcohol). There are also a considerable number of poisoning deaths involving multiple drugs [21]. Where co-proxamol is one of the drugs it is very likely to have been the lethal agent given its high toxicity. It is possible therefore that our findings underestimate the full effects of the withdrawal of co-proxamol. The increasing number of narrative verdicts recorded by coroners in England and Wales [22] could have influenced the findings through a small reduction in suicide verdicts, although this would probably have equally affected poisonings with co-proxamol and the other analgesics, and any impact would have been included in our analysis of suicide, open, and accidental deaths combined.

We have presented prescription data for the analgesics we have investigated. For some analgesics, notably paracetamol and co-codamol, sales will mainly be over-the-counter (OTC) rather than through prescription. Comprehensive OTC sales data are unfortunately not available; this would not, of course, affect the validity of the mortality data.

As noted above, we were unable to investigate the impact of the withdrawal on deaths involving morphine. However, most of such deaths result from illicit drug use [21].

Finally, we have not been able to assess possible compensatory increases in deaths from non-poisoning means that may have occurred following the withdrawal of co-proxamol. This is because of the range of other potential methods of suicide and also the likely temporal effects of other influences, such as the economic recession. However, since individuals often appear to show preference for particular methods of suicide [37], it is unlikely that any such effect would have been substantial.

### Conclusions

Withdrawal of co-proxamol in the UK appears to have had major beneficial effects on poisoning deaths involving this drug, including suicides and accidental poisonings, with no evidence of significant substitution by poisoning with other analgesics, in spite of their increased prescribing. Now that prescribing of the more toxic constituent of co-proxamol (dextropropoxyphene) has been withdrawn throughout Europe and production has ceased in the US and Canada the impact of this initiative should be evaluated on a larger scale.

## Supporting Information

Table S1 Interrupted time-series segmented regression analysis* of prescriptions (thousands) and mortality (number of deaths) of co-proxamol, other analgesics, all drugs, and all causes in England and Wales, 1998–2010.
(DOCX)

Text S1 Segmented regression analysis.
(DOCX)

## Abbreviations

CSM: UK Committee on Safety of Medicines
MHRA: UK Medicines and Healthcare products Regulatory Agency
NSAID: non-steroidal anti-inflammatory drug
ONS: Office for National Statistics
