# Supplementary material for: Six-Year Follow-Up of Impact of Co-proxamol Withdrawal in England and Wales on Prescribing and Deaths: Time-Series Study
Source: PLoS Med. 2012 May 8;9(5):e1001213. doi: 10.1371/journal.pmed.1001213 (PMC3348153; doi:10.1371/journal.pmed.1001213)
Supplement: Table S1 — Interrupted time-series segmented regression analysis* of prescriptions (thousands) and mortality (number of deaths) of co-proxamol, other analgesics, all drugs, and all causes in England and Wales, 1998–2010. (DOCX) [file pmed.1001213.s001.docx]

**Table S1**

Interrupted time series segmented regression analysis* of prescriptions (thousands) and mortality (number of deaths) of co-proxamol, other analgesics, all drugs, and all causes in England and Wales, 1998-2010

|  | **Regression Coefficients and Standard Errors (SE)** | | | | | | | | | | | |
| --- | --- | --- | --- | --- | --- | --- | --- | --- | --- | --- | --- | --- |
|  | ***Pre-intervention*** † | | | | | | ***Post-intervention*** † | | | | | |
|  | Base level | SE | P | Base trend | SE | P | Change in level | SE | P | Change in trend | SE | P |
|  | (β_0_) |  |  | (β_1_) |  |  | (β_2_) |  |  | (β_3_) |  |  |
| **Prescriptions** |  |  |  |  |  |  |  |  |  |  |  |  |
| Co-proxamol | 3263.058 | 350.294 | <0.001 | -55.438 | 13.813 | <0.001 | -581.900 | 48.575 | **<0.001** | 3.712 | 16.499 | 0.823 |
| Co-codamol | 1337.508 | 18.694 | <0.001 | 35.189 | 1.194 | <0.001 | 245.072 | 69.509 | **0.001** | 31.573 | 4.052 | **<0.001** |
| Codeine | 204.962 | 3.943 | <0.001 | 9.543 | 0.230 | <0.001 | 23.098 | 7.359 | **0.003** | 2.711 | 0.527 | **<0.001** |
| Co-dydramol | 1054.915 | 6.092 | <0.001 | -1.018 | 0.355 | 0.006 | 178.873 | 25.633 | **<0.001** | -9.476 | 1.499 | **<0.001** |
| Dihydrocodeine | 659.523 | 21.364 | <0.001 | -0.394 | 0.998 | 0.695 | -29.717 | 4.751 | **<0.001** | -2.703 | 1.570 | 0.092 |
| NSAIDs | 4655.803 | 47.083 | <0.001 | 28.125 | 3.000 | <0.001 | -707.122 | 68.453 | **<0.001** | -50.755 | 4.981 | **<0.001** |
| Paracetamol | 1488.220 | 99.268 | <0.001 | 42.798 | 5.124 | <0.001 | 94.470 | 61.795 | 0.133 | 34.323 | 7.741 | **<0.001** |
| Tramadol | 158.794 | 14.675 | <0.001 | 25.882 | 0.913 | <0.001 | 74.309 | 33.695 | **0.032** | 12.756 | 1.856 | **<0.001** |
| Other analgesics (excluding NSAIDS) ‡ | 4880.483 | 72.010 | <0.001 | 112.492 | 4.332 | <0.001 | 642.097 | 156.145 | **<0.001** | 64.849 | 10.046 | **<0.001** |
| Other analgesics ‡ | 9533.461 | 60.185 | <0.001 | 14.086 | 3.613 | <0.001 | -72.139 | 163.290 | 0.661 | 14.006 | 11.198 | 0.217 |
| **Deaths: Suicide and Open** |  |  |  |  |  |  |  |  |  |  |  |  |
| Co-proxamol | 70.111 | 3.478 | <0.001 | -0.890 | 0.239 | <0.001 | -15.558 | 4.932 | **0.003** | -0.430 | 0.331 | 0.201 |
| Co-codamol | 2.482 | 1.327 | 0.068 | 0.045 | 0.073 | 0.538 | 1.310 | 1.567 | 0.407 | -0.044 | 0.104 | 0.673 |
| Codeine | 0.913 | 0.542 | 0.099 | 0.096 | 0.052 | 0.072 | -0.167 | 1.403 | 0.906 | 0.016 | 0.101 | 0.875 |
| Co-dydramol | 1.753 | 0.302 | <0.001 | -0.022 | 0.017 | 0.206 | 1.229 | 0.653 | 0.066 | -0.075 | 0.038 | 0.054 |
| Dihydrocodeine | 9.474 | 2.281 | <0.001 | -0.107 | 0.113 | 0.346 | 1.069 | 1.512 | 0.483 | -0.007 | 0.129 | 0.958 |
| NSAIDs | 4.834 | 1.016 | <0.001 | -0.120 | 0.056 | 0.039 | 0.968 | 0.936 | 0.306 | 0.068 | 0.069 | 0.327 |
| Paracetamol | 30.002 | 2.341 | <0.001 | -0.323 | 0.139 | 0.025 | 2.289 | 2.837 | 0.424 | -0.097 | 0.179 | 0.592 |
| Tramadol | 0.908 | 0.557 | 0.110 | 0.127 | 0.032 | 0.000 | 0.378 | 1.192 | 0.752 | -0.116 | 0.074 | 0.125 |
| Other analgesics (excluding NSAIDs) ‡ | 45.471 | 3.236 | <0.001 | -0.173 | 0.199 | 0.388 | 5.426 | 4.710 | 0.255 | -0.297 | 0.282 | 0.298 |
| Other analgesics ‡ | 50.406 | 2.828 | <0.001 | -0.305 | 0.198 | 0.132 | 6.976 | 4.870 | 0.159 | -0.243 | 0.276 | 0.383 |
| All drugs except co-proxamol and other analgesics | 232.587 | 8.993 | <0.001 | -0.780 | 0.545 | 0.159 | -20.377 | 11.118 | 0.073 | 0.080 | 0.740 | 0.915 |
| All drugs | 351.810 | 12.579 | <0.001 | -1.890 | 0.848 | 0.031 | -31.642 | 18.168 | 0.088 | -0.592 | 1.161 | 0.612 |
| All causes | 1316.784 | 26.003 | <0.001 | -4.632 | 1.601 | 0.006 | -56.336 | 37.768 | 0.142 | 5.415 | 2.360 | **0.026** |
| **Deaths: Suicide, Open and Accidental** |  |  |  |  |  |  |  |  |  |  |  |  |
| Co-proxamol | 83.199 | 3.398 | <0.001 | -1.054 | 0.221 | <0.001 | -18.161 | 4.983 | **0.001** | -0.554 | 0.324 | 0.094 |
| Other analgesics (excluding NSAIDs) ‡ | 60.399 | 3.945 | <0.001 | -0.147 | 0.244 | 0.549 | 2.052 | 7.096 | 0.774 | 0.387 | 0.447 | 0.390 |
| Other analgesics ‡ | 66.354 | 3.942 | <0.001 | -0.279 | 0.258 | 0.285 | 2.401 | 7.677 | 0.756 | 0.488 | 0.462 | 0.295 |
| All drugs except co-proxamol and other analgesics | 428.311 | 13.917 | <0.001 | -2.274 | 0.967 | 0.023 | -24.940 | 21.317 | 0.248 | 8.740 | 1.681 | **<0.001** |
| All drugs | 578.543 | 18.273 | <0.001 | -3.647 | 1.289 | 0.007 | -39.643 | 27.992 | 0.163 | 8.658 | 2.091 | **<0.001** |

******* Regression based on equation (1).

† Intervention point is the end of 2004: the Committee for Safety of Medicines announcement in January 2005 on the withdrawal of co-proxamol

‡ Other analgesics: cocodamol, codeine, codydramol, dihydrocodeine, NSAIDS, paracetamol and tramadol
