# Supplementary material for: Six-Year Follow-Up of Impact of Co-proxamol Withdrawal in England and Wales on Prescribing and Deaths: Time-Series Study
Source: PLoS Med. 2012 May 8;9(5):e1001213. doi: 10.1371/journal.pmed.1001213 (PMC3348153; doi:10.1371/journal.pmed.1001213)
Supplement: Text S1 — Segmented regression analysis. (DOCX) [file pmed.1001213.s002.docx]

**Text S1 - Supporting Information**

**Segmented regression analysis**

The following is an explanation of the method of Wagner et al., [[27](#_ENREF_27)] applied to our particular analysis. Segmented regression analysis is a method of estimating changes in levels and trends in an outcome (prescribing and deaths, in our case) associated with an intervention (CSM announcement of the withdrawal of co-proxamol). The time series regression equation for this model is

*Ŷ_t_ = β_0_ + β_1_* x *time_t_ + β_2_* x *intervention_t_ + β_3_* x *time_after_intervention_t_ + e_t_* (1)

*Ŷ_t_* is the outcome (mean number of prescriptions or deaths per quarter); *time* indicates the number of quarters from the start of the series (1..52); *intervention* is a dummy variable taking the values 0 in the pre-intervention segment and 1 in the post-intervention segment; *time_after_intervention* is 0 in the pre-intervention segment and counts the quarters in the post-intervention segment at time *t* (1..24). The coefficient *β_0_* estimates the base level of the outcome (number of prescriptions or deaths) at the beginning of the series; *β_1_* estimates the base trend, i.e. the change in outcome per quarter in the pre-intervention segment; *β_2_* estimates the change in level of prescriptions (or deaths) in the post-intervention segment; *β_3_* estimates the change in trend in prescriptions (or deaths) in the post-intervention segment; *e_t_* estimates the error.

The model may be used to estimate the absolute effect of the intervention. This is the difference between the estimated outcome at a certain time after the intervention and the outcome at that time if the intervention had not taken place. For example, to estimate the effect of the intervention at the midpoint of the post-intervention period (when *time* = 40.5 and *time_after_intervention* = 12.5), we have

*Ŷ_40.5 (without CSM)_ = β_0_ + β_1_* x *40.5* (2)

*Ŷ_40.5 (with CSM)_ = β_0_ + β_1_* x *40.5 + β_2_ + β_3_* x *12.5* (3)

thus, the absolute effect of the intervention is

*Ŷ_40.5 (with CSM) –_ Ŷ_40.5 (without CSM) =_ β_2_ + β_3_* x *12.5* (4)

Coefficients and errors from full models including all terms in equation (1) are given in the Appendix table. Non-significant terms were included as there may be correlation between slope and level terms which should be accounted for.

The absolute effect of the intervention was calculated from equation (4), with standard errors calculated according to the method of Zhang et al. [[38](#_ENREF_38)]. The expression for the standard error includes the covariance between *β_2_* and *β_3,_* which for our calculation was obtained from an autoregression post-estimation command (estat vce) in Stata v10.0.
